# Progression-Free Survival with PARP Inhibitors According to Clinical Risk in Patients with Ovarian Cancer: An Indirect Comparison Using Reconstructed Data

**DOI:** 10.32604/or.2026.077700

**Published:** 2026-06-16

**Authors:** Lorenzo Gasperoni, Luna Del Bono, Alberto Farolfi, Andrea Messori, Vera Damuzzo

**Affiliations:** 1 Pharmaceutical Department, USL Toscana Centro, Prato, Italy; 2 Department of Pharmacy, School of Specialization in Hospital Pharmacy, University of Pisa, Pisa, Italy; 3 Medical Oncology, Breast & GYN Unit, IRCCS Istituto Romagnolo per lo Studio dei Tumori (IRST) “Dino Amadori”, Meldola, Italy; 4 HTA Unit, Regional Health Service, Firenze, Italy; 5 Italian Society of Clinical Pharmacy and Therapeutics (SIFaCT), Torino, Italy; 6 Hospital Pharmacy Department, Azienda Ulss 2 Marca Trevigiana, Treviso, Italy

**Keywords:** IPDfromKM, poly (ADP-ribose) polymerase (PARP) inhibitors, advanced ovarian cancer, indirect comparison

## Abstract

**Background:** Poly (ADP-ribose) polymerase (PARP) inhibitors (PARPi) are established maintenance treatments in ovarian cancer, but comparative efficacy across genetic profiles and relapse risk categories remains unclear. The aim of this study was to compare the efficacy of different PARPi as maintenance therapy in ovarian cancer across genetic profiles and relapse risk categories using reconstructed individual patient data (IPD) from randomized trials (RCTs). **Methods:** IPD were reconstructed using the IPDfromKM method from published Kaplan-Meier curves of RCTs stratified by clinical risk subgroup. Progression-free survival (PFS) was the primary endpoint. Three comparisons were performed: Breast Cancer gene (BRCA)+ high-risk, Homologous Recombination Deficiency (HRD)+/BRCAwt high-risk, and BRCA+ low-risk populations. Restricted Mean Survival Time (RMST) was calculated as a supplementary measure, with curves truncated at 66 months. **Results:** In the BRCA+ high-risk population, olaparib monotherapy (median PFS 41.2 months) and olaparib plus bevacizumab (median PFS 42.5 months) demonstrated the greatest PFS benefit, marginally outperforming niraparib (median PFS 31.2 months). RMST analysis showed a 14-month advantage for olaparib plus bevacizumab over bevacizumab alone. In the HRD+/BRCAwt high-risk population, olaparib plus bevacizumab and niraparib showed comparable efficacy, with no statistically significant inter-treatment difference. In the BRCA+ low-risk population, olaparib plus bevacizumab showed superior HR versus olaparib monotherapy, without reaching statistical significance. RMST analysis also indicated an advantage of 8.5 months for the combination, though this did not reach statistical significance. **Conclusions:** PARPi treatment benefit in ovarian cancer is meaningfully influenced by genetic profile and relapse risk, supporting biomarker-driven treatment selection in clinical practice.

## 1 Introduction

The advent of poly (ADP-ribose) polymerase (PARP) inhibitors has significantly reshaped the therapeutic landscape of ovarian cancer [1]. PARP inhibitors (PARPi) are currently the standard of care for first-line maintenance therapy. Furthermore, variability in therapeutic efficacy has been observed across patient subpopulations, underscoring the importance of Breast Cancer (BRCA) and Homologous Recombination Deficiency (HRD) gene status as critical biomarkers for patient stratification and evidence-based treatment selection.

The potential overlap in the use of PARPi prompted us to compare the results of five phase III PARPi studies [2,3,4,5,6] in first-line maintenance therapy for ovarian cancer, stratifying the results according to genetic profile [7]. The analysis was based on reconstructed individual patient data (IPD) of published Kaplan-Meier (KM) curves. We observed that olaparib-based regimes provide superior progression-free survival (PFS) benefit in BRCA-mutated patients, outperforming niraparib. In BRCA-wild-type/HRD-positive disease, olaparib plus bevacizumab yields moderate PFS gains over niraparib and rucaparib, while BRCA-wild-type/HRD-negative patients derive minimal benefit from PARPi comparable to bevacizumab alone. Similarly, in a recent systematic review and meta-analysis, Petousis et al. evaluated the efficacy and safety of first-line PARPi maintenance therapy for advanced-stage ovarian cancer patients based on their genetic signature and on the extent of residual disease (complete versus incomplete resection) [8].

Indeed, the risk of relapse and survival are influenced by several factors, such as surgical outcome, with improved overall survival (OS) observed in patients who achieve complete surgical resection compared with those who have residual disease after primary cytoreductive surgery [9]. For this reason, patients with stage III disease who had residual disease after upfront surgery, those who had received neoadjuvant chemotherapy, or who were metastatic at diagnosis were considered at higher risk. On the contrary, patients with stage III disease who underwent complete resection during initial surgery were considered to be at lower risk.

The results of Petousis et al. show that PARPi maintenance therapy was associated with improved PFS in patients with optimal cytoreduction (hazard ratio [HR], 0.51; 95% confidence interval [CI]: 0.44–0.60) and suboptimal cytoreduction (HR, 0.39; 95% CI: 0.25–0.62) and a reduced rate of any event in both populations. However, the 95% predictioninterval (PI) for the suboptimal cytoreduction group included the null (0.15–1.03). This result calls into question the effectiveness of PARPi maintenance therapy, which may have minimal impact on disease control in this high-risk subgroup. In another review, Reid-Schachter et al. evaluated the PFS benefits of PARPi therapies based on clinically relevant risk factors, including residual disease status and high-risk patient subgroups [10]. They emphasize that patients with visible residual disease after primary cytoreductive surgery experienced substantial advantages. These findings suggest an opportunity to refine the criteria used to select patients for PARPi therapy. However, current treatment guidelines for PARPi do not consider postoperative residual disease status or surgical timing in their treatment algorithms [10].

Recently, Lorusso et al. demonstrated in a post hoc analysis of the phase III PAOLA-1 trial that the combination of olaparib and bevacizumab for maintenance therapy should be considered the standard of care for all patients with newly diagnosed advanced ovarian cancer and HRD-positive tumors, regardless of their risk level for disease progression [11]. The analysis emphasized that five-year PFS rates were especially favorable for lower-risk patients.

In light of the new evidence highlighting the importance of risk stratification, we have updated our previous work and conducted an indirect treatment comparison using reconstructed IPD from published randomized controlled trials (RCTs) on the efficacy of PARPi maintenance in patients categorised by their genetic profile and risk of relapse. As we base our analysis on IPD, using the IPDfromKM method, the criterion for including or excluding the RCTs in the analysis is whether they have published KM curves that are stratified by clinical risk subgroup for BRCA+ patients and HRD+/BRCAwt patients. This work aims to make comparisons for all subgroups for which results from at least two clinical trials are available.

## 2 Materials and Methods

### 2.1 Literature Search

We conducted a search of the PubMed database (National Center for Biotechnology Information, Bethesda, MD, USA; https://pubmed.ncbi.nlm.nih.gov) to identify RCTs that were relevant to our analysis. The last search was performed on 30 September 2025. The search strategy was as follows: [(“Ovarian Cancer” OR “ovarian carcinoma”) AND (“Parp inhibitor” OR “PARPi” OR “parpi” OR Olaparib OR Niraparib OR Rucaparib OR Veliparib OR Senaparib OR Talazoparib OR Pamiparib) AND (“first-line” OR “first-line” OR maintenance OR “newly diagnosed”)]. The selection process adhered to the PRISMA guidelines [12]. The selection criteria were as follows: phase III RCT; first-line maintenance treatment of advanced ovarian cancer data presented through KM survival curves stratified by clinical risk subgroup. In addition, to ensure that no results from sub-analyses were excluded, further searches were performed for each RCT excluded solely due to the absence of stratified KM by risk group. These searches covered meeting abstracts and poster presentations from the American Societyof Clinical Oncology (ASCO), European Society of Medical Oncology (ESMO), European Society for Gynecologic Oncology (ESGO), and Society of Gynecologic Oncology (SGO). We excluded the studiesin which KM curves reported survival data starting not from the beginning of the maintenance phase, but also including the previous chemotherapy treatment.

### 2.2 Reconstruction of IPD

This work is a secondary statistical modeling study and indirect treatment comparison based on reconstructed IPD from published RCTs. For each selected RCT, IPD were reconstructed from the KM curves of the treatment and control arms using the IPDfromKM algorithm described by Liu et al. [13]. KM curves were digitized with WebPlotDigitizer (Ankit Rohatgi, Notre Dame, IN, USA; https://automeris.io/WebPlotDigitizer) (version 4.7; accessed October 2025; sampling parameters: distance = 20, Δx = 15, Δy = 15). The extracted X–Y coordinates, together with the reported numbers of enrolled patients and observed events, were then processed using the IPDfromKM package (version 1.2.3.0; last updated 22 March 2022). This procedure yielded a reconstructed dataset containing survival times—defined as the interval between trial enrollment and the last available follow-up—and event status, classified as alive/censored or dead/progressed. The resulting output provided reconstructed IPD for each arm of the included RCTs. The digitalization process and reconstruction of IPD were performed in duplicate by an independent researcher to grant reproducibility of results.

This analysis aimed to evaluate the relative efficacy of maintenance treatments with PARPi in patients with newly diagnosed advanced ovarian cancer after a first-line platinum-based chemotherapy. PFS is the primary endpoint, and we compared each risk group (low and high-risk) based on molecular expression (BRCA+, HRD+/BRCAwt). Patients with stage III disease who had undergone upfront surgery and had residual disease or who had received neoadjuvant chemotherapy, or who had stage IV disease, were considered at higher risk, and patients with stage III disease who had undergone upfront surgery and had complete resection were considered in the low risk group. The execution of the four potential sub-analyses (BRCA+ low-risk; BRCA+ high-risk; HRD+/BRCAwt low-risk; HRD+/BRCAwt high-risk) depends on the availability of published results with stratified KM curves for at least two clinical trials for each subgroup. Therefore, if the KM curve was not available in an RCT for one of the four subgroups, the RCT was excluded from analysis of that single subgroup.

KM curves of PFS were reconstructed for each trial arm. For comparative analyses, a placebo was adopted as the common comparator across the included trials. All IPD from placebo arms across the included studies were pooled and analyzed together to constitute the control group. As in the PAOLA-1 study, the control group consisted of an active treatment (bevacizumab), we assessed in each patient subgroup whether the use of bevacizumab induced the control group to behave differently from placebo-control arms. To assess this possible inconsistency of control groups across the included studies, the likelihood ratio test and the concordance statistic were employed, following established methods for evaluating inter-study heterogeneity. If heterogeneity was present, as in the BRCA+ high-risk population, the bevacizumab arm was considered separately as an active treatment.

### 2.3 Statistical Analysis

Treatment effects in comparison with control groups were estimated using the Cox proportional hazards model, with the results expressed as HR with 95% CI. In addition, indirect pairwise comparisons among active regimens were performed through the univariate Cox model. Restricted Mean Survival Time (RMST) was calculated as an additional measure of treatment effect for PFS, with KM curves truncated at 66 months, which corresponds to the minimum follow-up time available across the included RCTs. All statistical analyses were performed with R software (version 4.3.2).

## 3 Results

### Indirect Comparison Efficacy Analysis

**Figure 1:**
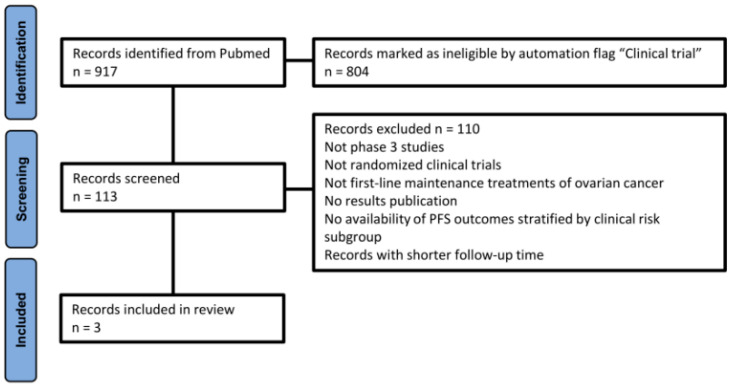
PRISMA flowchart of the process of trial selection.

A comprehensive literature search retrieved 917 PubMed (National Center for Biotechnology Information) records, which were screened based on predefined inclusion and exclusion criteria. The selection process, illustrated in Fig. 1 according to PRISMA recommendations, resulted in the identification of three RCTs eligible for indirect comparison analysis on PFS outcomes.

Results from the VELIA and FIRST trials were excluded because these studies reported PFS from the initiation of chemotherapy rather than from the start of PARPi maintenance therapy [14,15]. Consequently, their inclusion would have artificially prolonged PFS estimates relative to the other RCTs using the IPDfromKM method. For the ATHENA, PRIME, and FLAMES studies, we conducted additional searches for abstracts and posters [3,6,16]. Although we found two publications describing PFS in high and low-risk patients, the results were not presented using KM and they have consequently been excluded [17,18]. The three trials included in the analysis (PAOLA-1, SOLO1, PRIMA) enrolled patients with newly diagnosed ovarian cancer, mainly FIGO stage III or IV, who underwent primary or interval cytoreductive surgery and received either neoadjuvant or adjuvant chemotherapy with platinum-based regimens [11,19,20]. Main patients’ characteristics are summarized in Table 1.

**Table 1:** Summary of the main clinical characteristics of patients treated with PARPi included in the analysis.

Trial (Reference)	Treatments under Comparison	BRCA+ High Risk		BRCA+ Low Risk		HRD+/BRCAwt High Risk	
		N. of Pts	N. of Events PFS	N. of Pts	N. of Events PFS	N. of Pts	N. of Events PFS
SOLO1 (Banerjee et al., 2021 [20])	Olaparib	146	75	114	43	NA	NA
	Placebo	73	60	58	40	NA	NA
PAOLA1 (Lorusso et al., 2024 [11])	Olaparib+Bevacizumab	109	64	48	14	64	44
	Placebo+Bevacizumab	55	44	25	14	37	32
PRIMA (Monk et al., 2024 [19])	Niraparib	152	90	NA	NA	94	59
	Placebo	71	60	NA	NA	55	45

Note: N.: number; Pts: patients; BRCA: Breast Cancer gene; HRD: homologous recombination deficient; PFS: progression-free survival; N/A: not applicable.

The populations included in the studies are homogeneous in terms of histology and stage. The regimens evaluated included the PARPi olaparib, niraparib, as well as a treatment arm combining olaparib with bevacizumab. All studies compared the efficacy of PARPi to placebo, which was used as the common comparator in our analysis, except in the PAOLA-1 study; all patients enrolled in PAOLA-1 RCT also received bevacizumab alongside either PARPi or placebo during the maintenance phase.

The SOLO1 study only enrolled BRCA+ patients; therefore, the results can only be used to compare risk groups among BRCA+ patients [5]. The PAOLA-1 study enrolled patients regardless of their biomarker profile, so the stratified results can contribute to all subgroups’ analysis [2].

Enrollment into the PRIMA trial was restricted by surgical outcome, with lower-risk patients (R0) not eligible for inclusion, so the population was entirely high risk [4]. Thus, an indirect comparison of PRIMA patients with those in other selected RCTs will only concern high-risk categories. As there were three main subgroups present in the cohorts that could be compared based on risk group, BRCA and HRD status, we split our analysis into three parts.

The first comparison considers BRCA+ high-risk population from the PAOLA-1, SOLO1, and PRIMA studies, and a total of 606 patients were reconstructed. The second comparison considers BRCA+ low-risk population from the PAOLA-1 and SOLO-1 studies, and a total of 245 patients were reconstructed. The third comparison considers the HRD+/BRCAwt high-risk population from the PAOLA-1 and PRIMA studies, and a total of 250 patients were reconstructed. It was not possible to compare HRD+/BRCAwt low-risk population from the PAOLA-1 study with patients from any other study.

An analysis of heterogeneity was conducted to determine whether the control groups in the RCTs behaved similarly in terms of PFS. Results are reported in Supplementary Figs. S1–S3. The heterogeneity test revealed no substantial heterogeneity between the PFS of the control arms in the BRCA+ high-risk cohort (likelihood ratio test = 4.98 with two degrees of freedom, *p* = 0.08), in the HRD+/BRCAwt high-risk cohort (likelihood ratio test = 0.09 with one degrees of freedom, *p* = 0.8) and in the BRCA+ low-risk cohort (likelihood ratio test = 1.22 with one degrees of freedom, *p* = 0.3). However, in the BRCA+ high-risk cohort, the use of bevacizumab in the control arms of the PAOLA-1 trial conveys a certain degree of heterogeneity due to longer PFS compared to placebo-controlled RCT. Therefore, in the BRCA+ high-risk cohort, we opted to pool reconstructed IPD from the placebo arm of SOLO1 and PRIMA RCTs as the control arms and to consider the bevacizumab arm of the PAOLA-1 study as an independent treatment arm.

**Figure 2:**
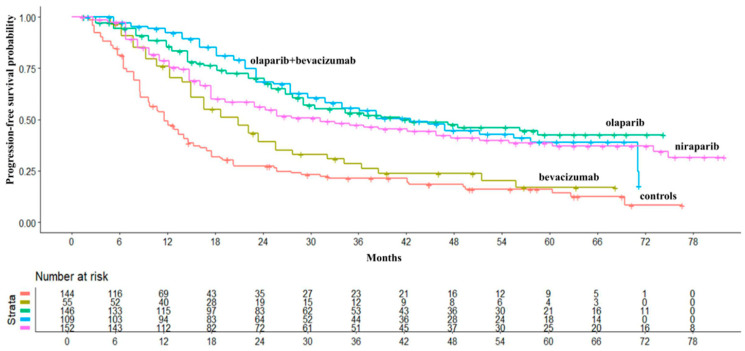
PFS of PARPi in BRCA+ high-risk population. After reconstruction of IPD from three trials, the following PFS KM curves were generated: olaparib+bevacizumab (n = 109; in light blue); olaparib (n = 146; in green); niraparib (n = 152; in pink); and bevacizumab (n = 55; in gold). The control arm (n = 144; in red) has been generated by pooling IPD from the control arms of the two trials (SOLO1 and PRIMA). Abbreviations: PFS: progression-free survival; PARPi: PARP inhibitors; BRCA: Breast Cancer gene; IPD: individual patient data; KM: Kaplan-Meier; n: number of patients.

In the BRCA+ high-risk population, maintenance therapy with PARPi significantly improved both HR and median PFS (Fig. 2, Table 2).

**Table 2:** HR with 95%CI for PFS of PARPi in BRCA+ high-risk population, HRD+/BRCAwt high-risk population, and BRCA+ low-risk population.

Population	Treatment	HR	Lower 95%	Upper 95%	*p*-Value
**BRCA+ high risk population**	**Bevacizumab**	0.66	0.46	0.95	0.025
	**Olaparib**	0.32	0.24	0.43	<0.001
	**Olaparib+bevacizumab**	0.35	0.26	0.48	<0.001
	**Niraparib**	0.41	0.31	0.54	<0.001
**HRD+/BRCAwt high risk population**	**Olaparib+bevacizumab**	0.56	0.38	0.82	0.003
	**Niraparib**	0.63	0.45	0.89	0.008
**BRCA+ low risk population**	**Olaparib**	0.37	0.24	0.57	<0.001
	**Olaparib+bevacizumab**	0.19	0.09	0.38	<0.001

BRCA: Breast Cancer gene; HR: hazard ratio; CI: confidence interval; HRD: Homologous Recombination Deficiency.

Indirect comparisons of the PFS benefits of different PARPi indicated that the combination of olaparib and bevacizumab and olaparib monotherapy are the most effective treatment for this patient subgroup (HR = 0.35; 95% CI: 0.26–0.48 *p* < 0.001 and median PFS of 42.5 months; 95% CI 31.77–71 for olaparib+bevacizumab; HR = 0.32; 95% CI: 0.24–0.43 *p* < 0.001 and median PFS of 41.2 months; 95% CI 29.09-NA for olaparib), with slightly higher efficacy compared to niraparib (HR = 0.41; 95% CI: 0.31–0.54 *p* < 0.001 and median PFS of 31.2 months; 95% CI 22.90–51.2). In the inter-treatment comparison, all therapeutic strategies involving a PARPi are superior to the bevacizumab control arm, but no PARPi (or combination) appears to be more effective than the others. The RMST analysis, which truncates the curve at 66 months, shows that olaparib+bevacizumab has an advantage of 14 months (51%) over bevacizumab alone (RMST 42.48 months; 95% CI 38.04–46.92 for olaparib+bevacizumab and RMST 28.0 months; 95% CI 22.19–33.80 for bevacizumab). Olaparib showed a RMST of 41.56 months (95% CI 37.37–45.76), confirming that the addition of bevacizumab to treatment with PARPi adds minimal benefit in this population. Niraparib showed the lower benefit among PARPi with a RMST of 37.22 months (95% CI 33.02–41.41). The relative increase in RMST gain was different between bevacizumab and PARPi: considering the RMST fold-increase bevacizumab showed an initial +46% increase in RMST between 18 and 36 months, while this gain was reduced to a + 29%. On the contrary PARPi showed a median increase of 62% within 36 months and continue to increase after 36 months (+50% RMST in the period between 36 and 66 months).

**Figure 3:**
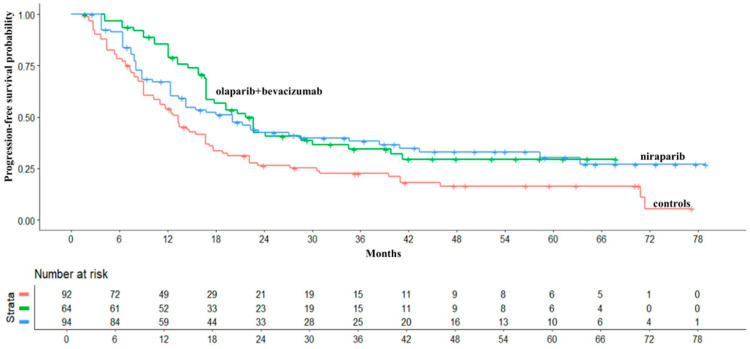
PFS of PARPi in HRD+/BRCAwt high-risk population. After reconstruction of IPD from two trials, the following PFS KM curves were generated: olaparib+bevacizumab (n = 64; in green); and niraparib (n = 94; in light blue). The control arm (n = 92; in red) has been generated by pooling IPD from the control arms of the two trials.

In the HRD+/BRCAwt high-risk population, olaparib+bevacizumab and niraparib monotherapy appear to have similar benefits in terms of HR (HR = 0.56; 95% CI: 0.38–0.82 *p* = 0.003 and HR = 0.63; 95% CI: 0.45–0.89 *p* = 0.008) (Fig. 3, Table 2) and the inter-treatment comparison confirms not significant HR (HR = 0.89; 95% CI: 0.53–1.49 *p* = 0.672).

The RMST analysis, which truncates the curve at 66 months, shows that both therapeutic strategies presented a similar RMST results (RMST 32.35 months; 95% CI 26.31–38.39 for olaparib+bevacizumab and RMST 31.16 months; 95% CI 25.76–36.56 for niraparib), 10 months longer than control arm (RMST 22.05 months; 95% CI 17.47–26.62). Notably, also in this population the relative increase in RMST gain was different between bevacizumab and PARPi: bevacizumab showed an initial +28% increase in RMST between 18 and 36 months, while this gain was reduced to a + 22%. On the contrary PARPi showed a median increase of 50% within 36 months and continued to increase after 36 months (+39% RMST in the period between 36 and 66 months).

**Figure 4:**
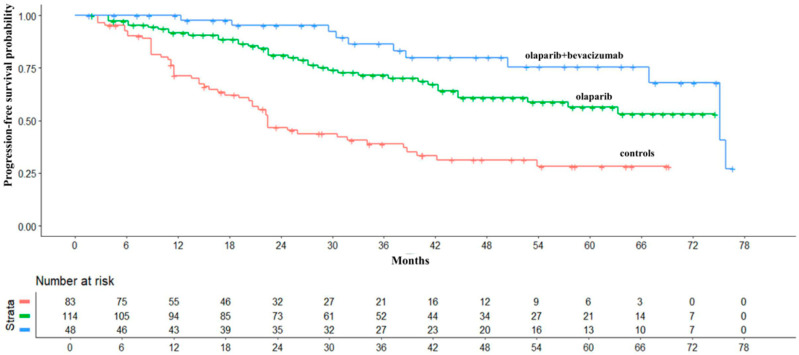
PFS of PARPi in BRCA+ low-risk population. After reconstruction of individual patient data (IPD) from two trials, the following PFS KM curves were generated: olaparib+bevacizumab (n = 48; in light blue); and olaparib (n = 114; in green). The controlarm (n = 83; in red) has been generated by pooling IPD from the control arms of the two trials. Abbreviations: n, number of patients.

In the BRCA+ low-risk population, maintenance therapy with PARPi significantly improved median PFS (Fig. 4, Table 2). The HR for olaparib+bevacizumab (HR = 0.19; 95% CI: 0.09–0.38, *p* < 0.001) was numerically lower compared with olaparib monotherapy (HR = 0.37; 95% CI: 0.24–0.57 *p* < 0.001), although not statistically significant in the inter-treatment comparison (HR = 0.50; 95% CI: 0.22–1.15 *p* = 0.100).

The RMST analysis highlights an advantage of 8.5 months for olaparib+bevacizumab (RMST 57.94 months; 95% CI 52.91–62.96) compared to olaparib monotherapy (RMST 49.41 months; 95% CI 45.04–53.77) and 25 months compared to the control arm (RMST 32.65 months; 95% CI 27.22–38.09).

## 4 Discussion

The advent of PARPi has profoundly transformed the therapeutic landscape of ovarian cancer. By exploiting the principle of synthetic lethality in tumors with defects in the homologous recombination repair (HRR) system, these agents have redefined the management of the disease, leading to a substantial improvement in long-term outcomes across several clinical settings. DNA damage repair (DDR) is a complex network encompassing multiple pathways responsible for maintaining genomic integrity [21]. To date, at least six major DDR pathways have been described, which coordinate the repair of single-strand breaks (SSBs) and double-strand breaks (DSBs). Among these, homologous recombination (HR) and non-homologous end joining (NHEJ) represent the principal mechanisms for DSB repair [22]. HR is an error-free process active during the S and G2 phases of the cell cycle, when a sister chromatid is available as a template, whereas NHEJ is a faster but error-prone mechanism that operates throughout most phases of the cell cycle. Deficiencies in HRR (HRD), frequently driven by alterations in genes such as BRCA1 and BRCA2, render tumor cells highly dependent on alternative repair pathways, thereby providing the biological rationale for PARP inhibition.

PARPi maintenance therapy is characterized by a favorable safety and tolerability profile, with most adverse events being manageable and reversible [23]. Beyond catalytic inhibition of PARP enzymes, these agents exert cytotoxic effects through the stabilization of PARP–DNA complexes, a phenomenon known as PARP trapping, which interferes with DNA replication and leads to the accumulation of DNA damage. The extent of PARP trapping varies among different PARPi and contributes to differences in their cytotoxic potency, with agents such as olaparib, niraparib, and rucaparib demonstrating significantly higher trapping efficiency compared with veliparib [24]. The most concerning toxicities—secondary malignancies such as myelodysplastic syndromes or acute myeloid leukemia—occur infrequently and do not significantly compromise the overall therapeutic index [25].

Although SOLO1, PAOLA-1, and PRIMA all established PARP inhibition as a cornerstone of maintenance therapy in newly diagnosed ovarian cancer, they differ markedly in design, patient population, and molecular enrichment. SOLO1 enrolled patients with BRCA1/2-mutated high-grade serous or endometrioid ovarian cancer who had achieved a complete or partial response after platinum-based chemotherapy, demonstrating an unprecedented improvement in PFS with olaparib monotherapy. PAOLA-1 extended this concept by evaluating olaparib in combination with bevacizumab among patients unselected for BRCA status but treated with first-line bevacizumab, showing a clear PFS benefit confined to HRD-positive tumors. In contrast, PRIMA assessed niraparib as maintenance in a broader, high-risk population regardless of BRCA or HRD status, confirming significant benefit across all subgroups, though most pronounced in BRCA-mutated and HRD-positive patients. Despite these methodological differences, collectively the three studies support HRD as the main predictive biomarker of PARP inhibitor benefit and underscore the lasting clinical relevance of upfront maintenance therapy in improving disease control. The pivotal phase III trials analyzed in this review, including SOLO1, PAOLA-1, and PRIMA, all assessed HRD status using the MyChoice^®^ test developed by Myriad Genetics. Building on these key studies, we demonstrated that the clinical benefit of PARPi in ovarian cancer maintenance therapy is highly dependent on both genetic profile and class of risk. Notably, while all therapeutic options in BRCA+ high-risk patients showed some efficacy, the modest RMST differences of 3–4 months between olaparib plus bevacizumab and olaparib suggest limited incremental benefit of dual PARP and VEGF inhibition, confirming that PARPi (olaparib or niraparib) are the leading treatments for these patients. In clinical practice, defining the optimal maintenance strategy for patients with BRCA wild-type ovarian cancer remains challenging. While HRD testing has allowed some refinement in therapeutic decision-making, its predictive accuracy is limited by variability in assay methodologies, cutoff definitions, and the biological complexity of homologous recombination proficiency. As a result, clinicians often face uncertainty in establishing whether PARPi, antiangiogenic agents, or sequential/combined approaches may offer the best long-term benefit for this heterogeneous group. To overcome these limitations, several functional and morphologic biomarkers are being investigated as complementary tools to guide treatment selection. In parallel, emerging high-throughput technologies such as proteomic profiling—including mass spectrometry–based approaches and protein array platforms—are enabling a deeper characterization of ovarian cancer biology by capturing dynamic changes in protein expression and signaling pathways. These approaches may provide additional insights into tumor behavior and adaptive responses to therapy, potentially identifying novel therapeutic vulnerabilities and mechanisms of resistance. The KELIM score, derived from early CA-125 kinetics, reflects platinum responsiveness and provides a dynamic estimate of chemosensitivity, while the Chemotherapy Response Score (CRS) offers a pathological measure of tumor regression after neoadjuvant treatment [26,27,28]. Preliminary evidence indicates that integrating these parameters could refine patient stratification beyond genomic profiling, enhancing the individualization of maintenance strategies [29].

In our study, we observed that the HRD+/BRCAwt high-risk population showed no meaningful advantage in terms of HR between olaparib+bevacizumab and niraparib, but a substantial difference was observed in terms of RMST over controls.

Notably, the comparable RMST values in placebo-only control arms across high-risk populations (21.47 months in BRCA+ vs. 22.7 months in HRD+) suggest that genetic background exerts minimal influence on natural disease progression in the absence of targeted therapy; however, this genetic determinism becomes clinically relevant upon treatment exposure, as all active therapeutic regimens demonstrate substantially greater RMST gains in the BRCA+ population, highlighting that the predictive—rather than prognostic—value of BRCA status emerges specifically in the context of DNA damage response-targeted interventions.

In high-risk populations, bevacizumab appears to produce an initial PFS advantage, with the survival curve widening compared to PARPi monotherapy. However, based on visual interpretation of the curves, this effect is limited to 24 months in the BRCA+ population and 18 months in the BRCAwt population. This trend could suggest the establishment of mechanisms of resistance to VEGF blockade. Future studies could focus on maintaining this advantage through the use of a VEGF inhibitor with greater resistance to escape mechanisms [30].

Coming to the low-risk population, the absence of KM curves for PFS in the HRD+/BRCAwt low-risk population hinders the possibility of having a complete scenario in low-risk patients. However, in the BRCA-mutated low-risk patients, the addition of bevacizumab to olaparib conveys more than 8 months of PFS gain in terms of RMST, which aligns with the synergistic mechanism of dual PARP and VEGF inhibition in tumors with pre-existing DNA repair defects. Our results, therefore, suggest that dual PARP and VEGF inhibition could lead to PFS benefits for both high-risk and low-risk patients, provided that early resistance is overcome.

In addition, in this subpopulation became evident that RMST analysis provides a more robust assessment than traditional hazard ratios, capturing the separation between the survival pattern of the KM curves.

Our approach employed KM curve digitization with individual patient data reconstruction, a technique with growing application in oncology and other medical fields. Prior validation work confirmed the high fidelity of our reconstruction process [31,32,33]. Key methodological strengths include retention of time-to-event information—typically omitted in standard meta-analyses using dichotomous outcomes—and generation of combined survival curves that facilitate direct visual comparison across interventions.

## 5 Limitation

Nevertheless, several limitations warrant consideration. The main limitations of this analysis arise from the indirect nature of the comparisons and from differences in trial design and patient selection across the included studies.

First, all efficacy estimates are based on reconstructed IPD derived from published KM curves, which may introduce approximation errors and cannot fully reproduce the original trial-level stratification factors. Moreover, the use of placebo as the common comparator relies on the assumption of exchangeability between control arms; although formal tests did not show significant heterogeneity in PFS, the systematic use of bevacizumab in the PAOLA-1 control arm likely contributed to longer PFS and a degree of clinical heterogeneity, particularly in the BRCA+ high-risk cohort where bevacizumab has been considered as a separate treatment arm rather than embedded into controls.

Second, important differences in eligibility criteria and baseline risk profiles limit the external validity of head-to-head inferences between PARPi strategies. SOLO1 enrolled exclusively BRCA+ patients with a favorable response to first-line platinum, PRIMA selected a uniformly high-risk population based on suboptimal debulking or neoadjuvant treatment, whereas PAOLA-1 included an unselected population treated with bevacizumab, with subsequent stratification by biomarker status. As a consequence, indirect comparison of PARPi therapies was hindered in some subgroups (for example, HRD-positive/BRCA wild-type low-risk patients) or it relies on a limited number of patients per group due to study design.

Third, the reliance on published KM curves for IPD reconstruction represents a methodological constraint that limited our ability to perform a comprehensive OS analysis, as not all included RCTs reported OS KM curves stratified by clinical risk subgroup. Indeed, the analysis focuses on PFS and RMST as surrogates of long-term benefit, without incorporating OS, subsequent lines of therapy, or safety outcomes, which may further influence the overall clinical value and generalizability of each maintenance strategy.

The results obtained should be considered hypothesis-generating rather than practice-changing. Although the analysis and methodology we propose are well-founded, they are affected by the above-described limitations, namely that indirect comparisons and reconstructed data inherently restrict causal inference.

Overall, our data suggest that maintenance with PARPi is effective in both high- and low-risk patients. However, the lack of KM curves prevented us from verifying the role of PARPi in HRD-/BRCAwt patients, where our previous results indicated modest benefits of these drugs with possible non-negligible toxic effects, even in the long term.

## 6 Conclusion

Despite the methodological limitations of indirect comparisons, this comparative analysis demonstrates that the advantages of PARP inhibition vary according to the patient’s genetic profile and risk of relapse. Recommendations for PARPi-based treatment and the potential usefulness of combining it with bevacizumab should therefore take these characteristics into account.

Our study confirms the need for further evidence to support the use of PARPi-based regimens in patients with ovarian cancer, and to improve clinical guideline recommendations.

## Data Availability

All data supporting the results of this study are included within the article and/or its Supplementary Materials.

## Supplementary Materials

The supplementary material is available online at https://www.techscience.com/doi/10.32604/or.2026.077700/s1. Supplementary Figure S1: Kaplan–Meier curves of PFS generated after reconstructing patient-level data from the placebo-treated control arms in BRCA+ high-risk cohort of the included trials. PAOLA1 (n = 55; in blue), PRIMA (n = 71; in green), and SOLO1 (n = 73; in red). Supplementary Figure S2: Kaplan–Meier curves of PFS generated after reconstructing patient-level data from the placebo-treated control arms in HRD+/BRCAwt high risk cohort of the included two trials. PAOLA1 (n = 37; in red) and PRIMA (n = 55; in light blue). Supplementary Figure S3: Kaplan–Meier curves of PFS generated after reconstructing patient-level data from the placebo-treated control arms in BRCA+ low risk cohort of the included two trials. PAOLA1 (n = 25; in light blue), and SOLO1 (n = 58; in red).
