# Supplementary material for: Progression-Free Survival with PARP Inhibitors According to Clinical Risk in Patients with Ovarian Cancer: An Indirect Comparison Using Reconstructed Data
Source: Oncol Res. 2026 Jun 16;34(7):14. doi: 10.32604/or.2026.077700 (PMC13292047; doi:10.32604/or.2026.077700)
Supplement: Supplementary file 1 [file OncolRes-34-77700-s001.zip › TSP_OR_77700-s001.pdf]

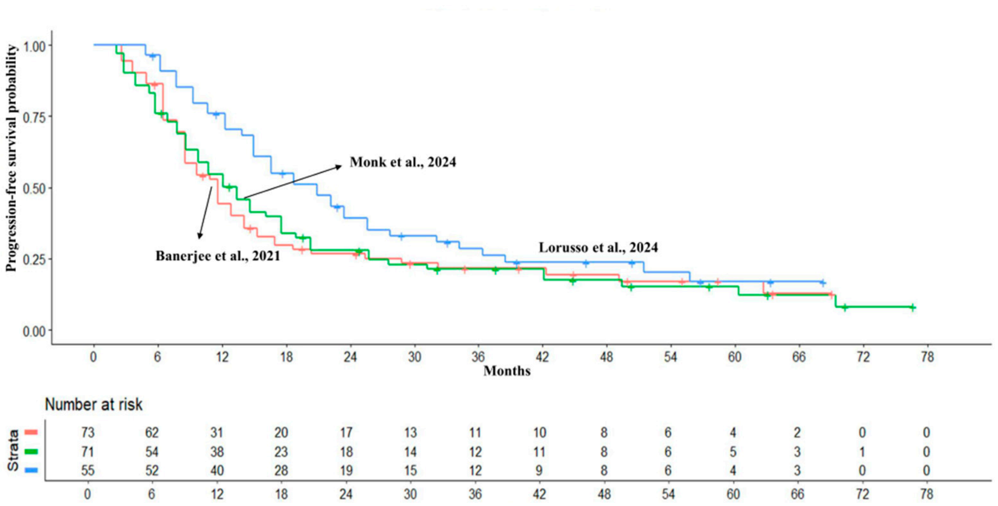

Supplementary Figure S1. Kaplan–Meier curves of PFS generated after reconstructing patient-level data from the placebo-treated control arms in BRCA+ high-risk cohort of the included trials. PAOLA1 (n = 55; in blue), PRIMA (n = 71; in green), and SOLO1 (n = 73; in red). Endpoint: progression-free survival (PFS), time in months. Abbreviations: PFS, Progression-free survival; BRCA: BReast CAncer gene; n, number of patients.

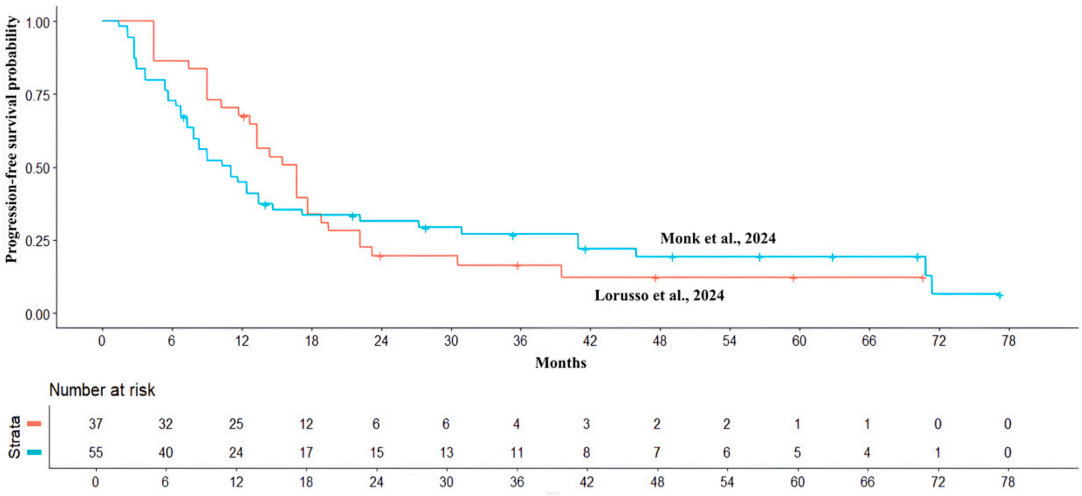

Supplementary Figure S2. Kaplan–Meier curves of PFS generated after reconstructing patient-level data from the placebo-treated control arms in HRD+/BRCAwt high risk cohort of the included two trials. PAOLA1 (n = 37; in red) and PRIMA (n = 55; in light blue). Abbreviations: HRD, Homologous Recombination Deficiency.

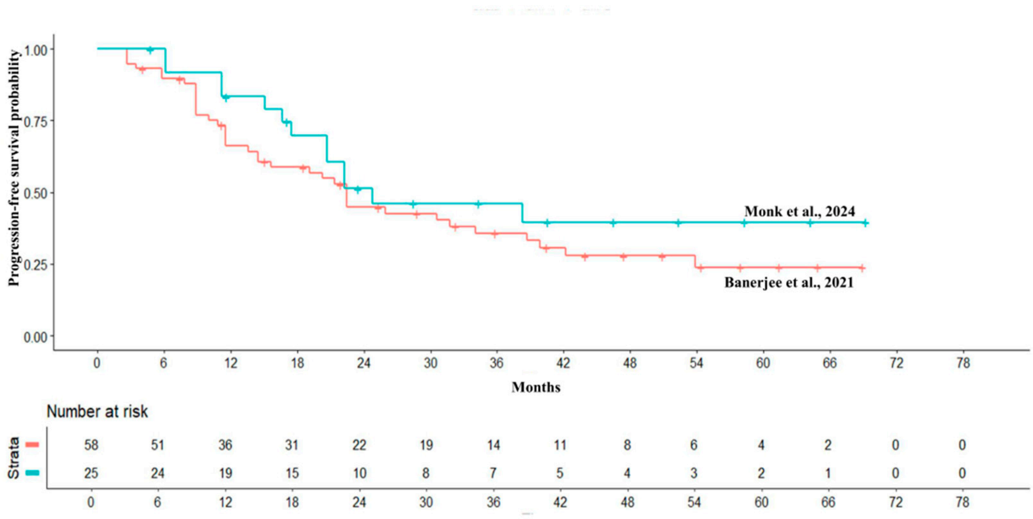

Supplementary Figure S3. Kaplan–Meier curves of PFS generated after reconstructing patient-level data from the placebo-treated control arms in BRCA+ low risk cohort of the included two trials. PAOLA1 (n = 25; in light blue), and SOLO1 (n = 58; in red).
